# MSMpred: interactive modelling and prediction of individual evolution via multistate models

**DOI:** 10.1186/s12874-023-01951-3

**Published:** 2023-05-24

**Authors:** Leire Garmendia Bergés, Jordi Cortés Martínez, Guadalupe Gómez Melis, DIVINE project

**Affiliations:** Department of Statistics and Operations Research, Universitat Politècnica de Catalunya, Barcelona, Spain

**Keywords:** Shiny app, Multistate models, COVID-19

## Abstract

**Background:**

Modelling the course of a disease regarding severe events and identifying prognostic factors is of great clinical relevance. Multistate models (MSM) can be used to describe diseases or processes that change over time using different states and the transitions between them. Specifically, they are useful to analyse a disease with an increasing degree of severity, that may precede death. The complexity of these models changes depending on the number of states and transitions taken into account. Due to that, a web tool has been developed making easier to work with those models.

**Results:**

MSMpred is a web tool created with the shiny R package that has two main features: 1) to allow to fit a MSM from specific data; 2) to predict the clinical evolution for a given subject. To fit the model, the data to be analysed must be upload in a prespecified format. Then, the user has to define the states and transitions as well as the covariates (e.g., age or gender) involved in each transition. From this information, the app returns histograms or barplots, as appropriate, to represent the distributions of the selected covariates and boxplots to show the patient’ length of stay (for uncensored data) in each state. To make predictions, the values of selected covariates from a new subject at baseline has to be provided. From these inputs, the app provides some indicators of the subject’s evolution such as the probability of 30-day death or the most likely state at a fixed time. Furthermore, visual representations (e.g., the stacked transition probabilities plot) are given to make predictions more understandable.

**Conclusions:**

MSMpred is an intuitive and visual app that eases the work of biostatisticians and facilitates to the medical personnel the interpretation of MSMs.

## Background

A multistate model (MSM) generalizes the classic survival modelling allowing the description of complex dynamical processes over time. It is defined by a series of states — which could represent different stages of the life history of an individual — and the transitions that connect them — which describe the potential paths between those states along time [1].

As examples in the context of COVID-19, Ursino et al. [2] and Mody et al. [3] used MSMs in order to describe the evolution of patients admitted to the Intensive Care Unit (ICU), and hospitalized patients, respectively. They consider several states, such as inpatient floor, ICU and invasive mechanical ventilation (IMV). The conclusions raised from these papers are powerful because they are able to estimate the factors associated to each transition and not only those that focus on the discharge or death.

Since these type of models are usually complex, different tools have been developed to ease the modellization process and to help with the interpretability of the findings. For instance, the app **MSMshiny** [4] aims to fit multistate models. It allows the user to: i) add an initial state even if it is not included in the uploaded data; ii) include as many transitions as desired; iii) assign specific covariates to each transition; iv) specify different models and compare their log-likelihood and Akaike information criterion (AIC) to help in selecting the best one. Furthermore, MSMshiny provides an interactive visualization of how individuals move between states over time. Among the main limitations of this app, we highlight that once a transition has been defined, it cannot be removed anymore and that the model cannot be validated through a residual analysis. Despite MSMshiny allows to make predictions, the outputs are limited and they do not help in the interpretation of the results. The app **MSMplus** [5] is intended to offer a wide spectrum of visualizations once an MSM is fitted outside the application. It provides a nice friendly interface, which provides various types of graphics, together with some indications, allowing a thorough interpretation of the fitted model. MSMplus allows a deep customization of the returned plots (e.g., changing the names of the states) and is complemented with theoretical explanations on MSMs. A weakness of the MSMplus app is that the users have to perform the analyses on their own and upload the outputs to obtain a complete data visualization of the results. In addition, the app lacks some numerical output, such as a table with the estimates of the fitted model. The **MSDshiny** app [6] has been designed specifically for clinical trials allowing the user to make simulations based on MSMs. The total number of states is decided by the user in the model specification. The simulation of the time-to-event times is performed using a Weibull distribution with specific parameters for each transition. Some of its main limitations are the lack of the relevant transition probability plots, the restriction to a maximum of five states and that it does not allow having transient initial states.

This work presents the **MSMpred** shiny app (https://www.grbio.eu/pubs/MSMpred/) designed to fit a MSM from specific data and to predict the clinical evolution for a given patient based on the previous model. We aim to overcome the above mentioned limitations of the existing apps. Before presenting **MSMpred** we provide an overview of MSM.

### Multistate models

A MSM is a model for a continuous time stochastic process allowing individuals to move among a finite number of states. Within the scope of survival analysis, MSMs allow to describe complex clinical processes that change over time. Those models are formed by states and transitions, which represent, for instance, the different stages of a disease evolution and the possible paths to move between those states, respectively. In these models we focus on the transitions between states and the time until they occur.

Usually those models are represented using a diagram where the nodes represent the states of the model, and the arrows represent the transitions. The diagram provides a visualization of the model allowing a complete understanding of all possible trajectories.

There are three different types of states: initial states are the ones where an individual could start the process; transient states are those in which individuals can get in and out of the state; and absorbing states are the ones where the process ends.

The main goals of MSMs are to characterize the process of an individual or a group; to analyse the relationship between the covariates of interest and the process transitions; to identify the risk factors for specific transitions; and to develop predictive models for new individuals.

There are three main steps to build a MSM: 1) represent the clinical process by means of states and transitions; 2) decide which covariates or factors are considered in each transition; 3) fit the model. The first two steps usually require a clinical insight; so, collaboration with medical experts is essential.

#### Characterization

There are different ways to characterize a MSM, related to each other in such a way that one characterization can be obtained from any of the others.

Let $X=\{X(t): t\ge 0\}$ be the stochastic process where *X*(*t*) represents the state in which the subject is at time *t* and takes values in the discrete set of states of the model, $\mathcal {R}=\{1,..,R\}$. The class $\mathcal {H}(t) = \{X(s), {\textbf {Z}}(s), s \le t\}$ contains the information of all the paths of all the individuals up to time t including the covariates ${\textbf {Z}}(s)$, which might be time-dependent.

The Markov property is met when, for any $s<t$ and $l,k \in \mathcal {R}$,

1 $$\begin{aligned} \Pr \{X(t)=l|X(s)=k;\mathcal {H}(s^-)\}{} & {} \nonumber \\ =\Pr \{X(t)=l|X(s)=k\}{} & {} \end{aligned}$$

where $\mathcal {H}(s^-)$ represents the history of the process prior to time *s*. This condition implies that the future path of a subject only depends on the state where he/she is at present but not on the past path that he/she had followed. Under the Markov assumption an MSM process can be characterized by means of either transition probabilities, transition intensities and cumulative transition intensities that we define next [7].

**Transition probability**, $\pi _{kl}(s,t)$: the probability that a subject in state *k* at time *s* ($s < t$) is in state *l* at time *t*, that is,

2 $$\begin{aligned}{} & {} \pi _{kl}(s,t)=\Pr \{X(t)=l|X(s)=k\}, \;\; \forall k \ne l \in \mathcal {R},\nonumber \\{} & {} \pi _{kk}(s,t)=1-\sum _{k \ne l}\pi _{kl}(s,t), \;\; \forall k \in \mathcal {R}. \end{aligned}$$

**Transition intensity**, $\lambda _{kl}(t)$: the transition intensity represents the instantaneous probability of transition between two states, *k* and *l*, at a specific time point *t* and it is defined as

3 $$\begin{aligned} \lambda _{kl}(t)= & {} \lim _{\Delta t\rightarrow 0} \frac{\Pr \{X(t+\Delta t)=l|X(t)=k\}}{\Delta t}\nonumber \\= & {} \lim _{\Delta t\rightarrow 0} \frac{\pi _{kl}(t,t+\Delta t)}{\Delta t}, \;\; \forall k \ne l \in \mathcal {R},\nonumber \\ \lambda _{kk}(t)= & {} -\sum _{k \ne l} \lambda _{kl}(t), \;\; \forall k \in \mathcal {R}. \end{aligned}$$

**Cumulative transition intensity**, $\Lambda _{kl}(t)$: the cumulative transition intensity between states *k* and *l* is defined as

4 $$\begin{aligned} \Lambda _{kl}(t)=\int _0^t \lambda _{kl}(s) ds, \;\; \forall k,l \in \mathcal {R}. \end{aligned}$$

#### Estimation

A non-parametric estimation of the cumulative transition intensities is given by the **Nelson-Aalen estimator** [7]. This estimator is based on the number of direct transitions $k \rightarrow l$ before time *t*, denoted by $N_{kl}(t)$, and the number of individuals in state *k* just before time *t*, denoted by $Y_k(t)$. Then, the Nelson-Aalen estimator for the cumulative transition intensity is given by

5 $$\begin{aligned} \hat{\Lambda }_{kl}(t)= & {} \int _0^t \frac{dN_{kl}(s)}{Y_k(s)} ds, \;\; \forall l \ne k \in \mathcal {R}\nonumber \\ \hat{\Lambda }_{kk}(t)= & {} -\sum _{k \ne l} \hat{\Lambda }_{kl}(t), \;\; \forall k \in \mathcal {R}. \end{aligned}$$

Semi-parametric regression models are very convenient to analyse MSMs as they are very flexible. This version of **MSMpred** only includes the Cox proportional hazards model. Since a Cox model is fitted for every transition, different subsets of covariates might be chosen to indicate their influence on different states of the model.

The **Cox or proportional hazards model** allows to relate the characteristics of an individual (described by some covariates) and the transition intensities:

6 $$\begin{aligned} \lambda _{kl}(t;{\textbf {Z}}) = \lambda _{kl,0}(t)\exp (\varvec{\beta }_{kl}^T {\textbf {Z}}) \end{aligned}$$

where $\lambda _{kl,0}(t)$ is the baseline intensity function for the transition $k \rightarrow l$, $\varvec{\beta }_{kl}$ is the vector of regression parameters, and ${\textbf {Z}}=(Z_1,...,Z_p)$ is the covariate vector.

The association of a covariate $Z_m$ with the transition intensity of a specific transition $k \rightarrow l$ can be measured by means of the **hazard ratio** ($\text {HR}$):

7 $$\begin{aligned} \text {HR}_{kl,q} = \exp (\beta _{kl,q}). \end{aligned}$$

The Cox model is built under three assumptions that need to be validated for each transition after every preliminary fitting. The assumptions are the following: first, the continuous covariates must act linearly on the logarithm of the hazard ratio; second, the final fitted model must lack influential observations; and third, the hazard rates between every two values of each covariate must be proportional. If any of these assumptions does not hold for a specific transition, the corresponding subset of covariates has to be changed and the model has to be readjusted [8]. **MSMpred** allows to make all these validations graphically using several types of residuals. In this app we have implemented the following residuals:

**Martingale-based residuals** in order to validate the linearity in the continuous covariates. A graph representing the martingale-based residuals of the selected transition and covariate as function of covariate values is reported. If the smoothed curve along the x axis is reasonably linear, we can assume the linearity of that covariate in that specific transition, otherwise, a transformation of the covariate in that specific transition should be implemented.

**Residuals based on the scores** to validate the global fit and to detect influential individuals. The user can plot the dfbetas residuals versus each covariate for any specific transition. The dfbetas residuals are the standardized dfbeta residuals which give a measure of the approximate change of the coefficients if the individual *i* is not taken into account [9]:

8 $$\begin{aligned} r_{df_{i,kl}}(t) = \hat{\varvec{\beta }}_{kl} - \hat{\varvec{\beta }}_{kl(i)}, \end{aligned}$$

where $\hat{\varvec{\beta }}_{kl}$ represents the estimator obtained when adjusting the Cox model for the transition $k \rightarrow l$ considering all the individuals, and $\hat{\varvec{\beta }}_{kl(i)}$ the estimator from the model without taking into account the individual *i*. These graphs help to detect if there is any potential influential value. Since large values of dfbetas indicate observations that are influential in estimating a given parameter, the larger the value of dfbetas the more influential the observation is.

**Schoenfeld residuals** to validate the proportional hazards premise. These residuals show the difference between the observed value of a covariate for a given selected transition and for every failure and the expected value under proportionality of the hazards. A graph with the scaled residuals over time can be plotted for each covariate. Each of these graphs should be independently assessed, and if the confidence band of the smoothed curve covers the line $r_{SC_{kl,m}}=0$, the proportionality of the hazard of covariate $Z_m$ in the transition $k \rightarrow l$ is not severely violated.

#### Prediction

As we will use MSMs to make predictions for new individuals, a predictive model needs to be specified to obtain $\tilde{\text {P}}\{X(t_1)=x|\mathcal {H}(t_0)\}$, where $t_1>t_0$ and $\mathcal {H}(t_0)=\{\mathcal {X}(t_0), \mathcal {Z}(t_0)\}$ is the observed history of states and covariates until time $t_0$. The tilde over P indicates that this is the predicted probability and not the observed probability, $\Pr \{X(t_1)=x|\mathcal {H}(t_0)\}$. Once the predictive model is obtained, the transition probabilities and hazard functions could be obtained in order to forecast different aspects of interest (e.g., probability of being in each state after time *t*).

To assess how good are those predictions, we need to analyse the calibration and sharpness of the predictive model. Concerning the calibration analysis the predicted probabilities, $\tilde{\text {P}}$, and the true probabilities, *P*, are compared, aiming to check how near/far are from each other. Ultimately systematically biased predictions can be detected [7]. With the sharpness of the model we analyse if the baseline information given by $\mathcal {H}(t_0)$, has a highly predictive value for the state where individuals will be later in time.

Some scoring rules, such as the logarithmic score, combine both aspects, calibration and sharpness, to analyse the performance of the predictive models. The logarithmic score for a group of *n* individuals is computed as

9 $$\begin{aligned} \text {LS}(\tilde{\text {P}}, t_1) = -\frac{1}{n}\sum _{i=1}^n \log \tilde{\text {P}}\{X_i(t_1)=x_i(t_1)| \mathcal {H}_i(t_0)\} \end{aligned}$$

and takes values between 0 and $\infty$. This score is useful to compare different models, where models with lower values of $\text {LS}(\tilde{\text {P}}, t_1)$ are preferable. The value of the the logarithmic score by itself is not, however, interpretable.

The article is from now on structured as follows. It starts with an Implementation section introducing the different tabs of the **MSMpred** describing their inputs and outputs, together with some indications about how to interpret them. For illustrative purposes the app is accompanied by data from a subset of four cohorts corresponding to four different Spanish waves of the COVID-19 pandemic. This dataset (uploaded on July 28th, 2022) has information about 4,000 hospitalized adult COVID-19 patients from 8 Catalan hospitals and it is part of the *Dynamic evaluation of COVID-19 clinical states and their prognostic factors to improve the intra-hospital patient management* project (DIVINE project, 2020PANDE00148, https://grbio.upc.edu/en/research/projects/pandemies). This project was approved by the Ethics Committee of the Hospital Universitari de Bellvitge. The Results section uses this dataset to illustrate the different capabilities of the app, which is open to further implementations outlined in the Discussion section. This paper ends by listing the future work of **MSMpred** and comparing the app to other web tools related to MSMs.

## Implementation

**MSMpred** is a shiny app with two main goals: 1) to fit a MSM from specific data; 2) to predict the clinical evolution for a given individual based on a previously fitted MSM. The user can upload a new dataset, provided that it has the required format explained in the help of the app.

As **MSMpred** is mainly designed for physicians or researchers with little knowledge about MSMs or statisticians that want to analyse data in a quick and visual way, the app is very friendly, implementing all the statistical part in an intuitive way and including interpretations for the different outputs.

### Software

This app has been created using shiny (version 1.7.3), an R package to create interactive web applications. The user can access this app either using the link (https://www.grbio.eu/pubs/MSMpred/) or downloading the R code that is available in github (https://github.com/LeireGarmendia/MSMpred) and locally running it. Other R packages used to implement the app and to improve the user interface are shinyBS, shinyWidgets, shinydashboard, shinydashboardPlus, shinyalert, shinyMatrix, and shinyjs. For implementing the MSM the app uses the mstate package following the indications given by Wreede [10]. The plots are made using the packages ggplot2, bshazard, cmprsk, DiagrammeR, LoopDetectR, survminer, pals, and the tables using DT and summarytools. Finally, another packages for data manipulation, such as dplyr, stringr, and lubridate are needed. The app was deployed using the version 4.2.1 of the R statistical software (https://cran.r-project.org/bin/windows/base/old/4.2.1/).

**MSMpred** has different sections identified by tabs where the user defines different aspects of MSMs (e.g., selection of the covariates or characteristics of the new individual) using parameterizable inputs. They are shortly described below along with their features:

- **Home**: app explanation, example data description and required format for data.
- **Data**: dataset upload, state and covariate labels customization and filters to apply to the dataset. Descriptive analysis of the covariates.
- **Model specification**: states and transitions definition and covariate selection for each transition.
- **Exploring the data**: descriptive and non-parametric plots related to MSM.
- **Model output**: selection of the type of model to be fitted, model output by means of tables and forest-plots, model validation and model comparison.
- **Predictions**: predictions for new individuals based on their characteristics.

## Results

Hereafter we explain a case study using the example dataset with the aim of showing the capability of MSMpred. Our goal is to show how the app works and its potential, not to reach conclusions from the results.

**Fig. 1:**
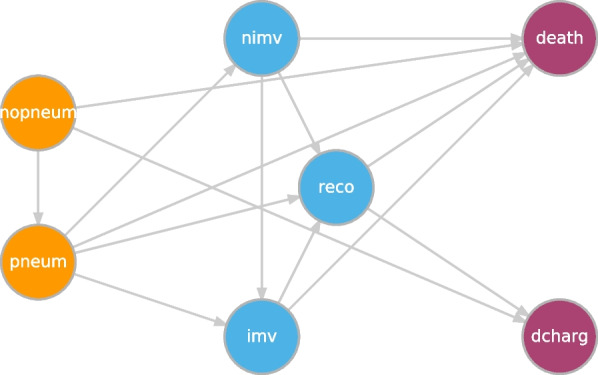
Multistate model of the DIVINE project

The example data consists of 3,984 hospitalized adult COVID-19 patients from 8 Catalan hospitals during 4 different waves (1st, 2nd, 3rd and 5th) of the pandemic in Spain. The default MSM consists of 7 states and 14 transitions (Fig. 1). Those states are: 1) **No severe pneumonia** *(nopneum)*: patients without severe pneumonia and without mechanical ventilation; 2) **Severe pneumonia** *(pneum)*: patients with severe pneumonia defined as fraction of inspired oxygen (Fi02) requirements $> 35\%$ but no need for mechanical ventilation [11]; 3) **Recovery** *(reco)*: patients recovered but still hospitalized; 4) **Non-invasive mechanical ventilation** *(nimv)*: patients with non-invasive mechanical ventilation (i.e. Optiflow and/or Bilevel positive airway pressure dispositives); 5) **Invasive mechanical ventilation** *(imv)*: patients with invasive mechanical ventilation (i.e., endotracheal intubation); 6) **Discharge** *(dcharg)*: patients that go home or to another hospital after recovering from COVID-19; 7) **Death** *(death)*: patients that die in the hospital due to COVID-19. The covariates used in this example are **wave** (with categories 1, 2, 3 and 5); **sex** (*Man*/Woman); **age** (age in years at hospital admission); **cardiovascular diseases (*card_vasc*)** (*No*/*Yes*); **immunosuppressed (*immune*)** (*No*/*Yes*); **vaccinated (*vacany*)** (*No*/*Yes*); **Charlson index (*charlson*)** (comorbidity index at hospital admission: it ranges from 0 to 24) [12]; **PaO2/FiO2 (*safi*)**; **severity score for community-acquired pneumonia (*curb65*)**; **pneumonia severity index (*psi*)** (index to measure the mortality for adults with community-acquired pneumonia); **C-reactive protein (*crprot*)** (it checks for inflammation in the body, usually caused by an infection. Normal values range from 0.8 to 3.0 mg/L); and **lymphocytes (*lympho*)** (number of $10^3$ lymphocytes per $\text {mm}^3$: in normal conditions it ranges from 1 to 4.8 cells/$\text {mm}^3$).

### Data

The user can upload a new dataset, provided that it has the required format. The app allows to filter the data by some covariates and to work with a subset of the original data. An example dataset from the DIVINE project is available and for our case study, we filter the data to only consider patients from the fifth Spanish wave of the pandemic (July-August 2021, $n=690$).

The names of the states and covariates can be customized in the numerical and graphical outputs. To check if the data has been imported correctly and as a first exploration of the data, descriptive plots and tables of the covariates are shown to observe their distribution and their main summary statistics.

### Model specification

The first step is to define the model by specifying the states and transitions of the desired MSM. From the names of the columns of the dataset, the app identifies what are the states, and by drop-downs menus the user can select the transitions between them. It is important to only include those transitions relevants for the problem and with enough individuals passing through them so that the estimation of the associated parameters is feasible. In general, at least 5 events per covariate are needed in each transition, otherwise it is not possible to estimate the corresponding parameters [13]. On the other hand, if one specific state does not need to be in the model, it is enough not to include it as part of any transition.

**Fig. 2:**
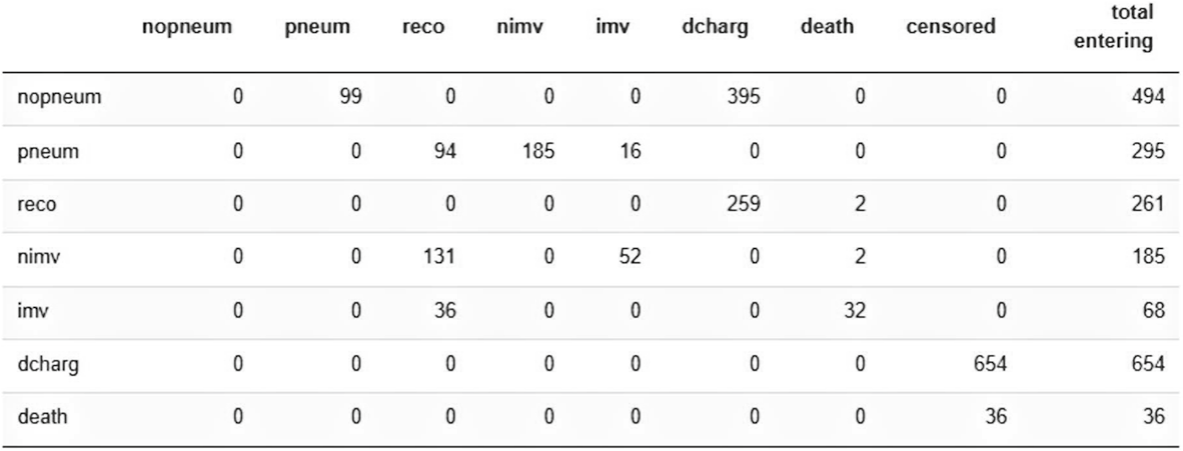
Number of individuals per transition for the DIVINE MSM

Figure 1 shows the diagram of the defined model where there are two orange initial states (*nopneum, pneum*), three non-initial blue transient states (*reco, nimv, imv*) and two purple absorbing states (*death, dcharg*). The colors help to distinguish the different roles that each state plays into the model. Figure 2 reveals the number of events for each transition, which is helpful to decide whether or not a specific transition should be included and to know how many covariates could be included in each transition [14]. The number of events and individuals per transition are the same because **MSMpred** does not allow the inclusion of loops into the model that would permit an individual to pass twice through the same transition.

In the proposed﻿ model there are four transitions that have less than five individuals: *nopneum* $\rightarrow$ *death*, *pneum* $\rightarrow$ *death*, *reco* $\rightarrow$ *death* and *nimv* $\rightarrow$ *death*. Due to their small sample size we will not consider those transitions and we removed them from the model.

The *covariates/time specification* tab contains a table to be filled with potential covariates in columns and the transitions in rows to specify covariates for each transition. In this same tab, the follow-up time to be considered in the graphical representations and in the predictions can be specified. In our example, the follow-up time is fixed to 30 days, the period for which we are interested in the patient’s evolution.

Once all the model specification is finished, it is mandatory to click on the *save model specification* button to be applied in subsequent sections of the app.

### Exploring the data (EDA)

**Fig. 3:**
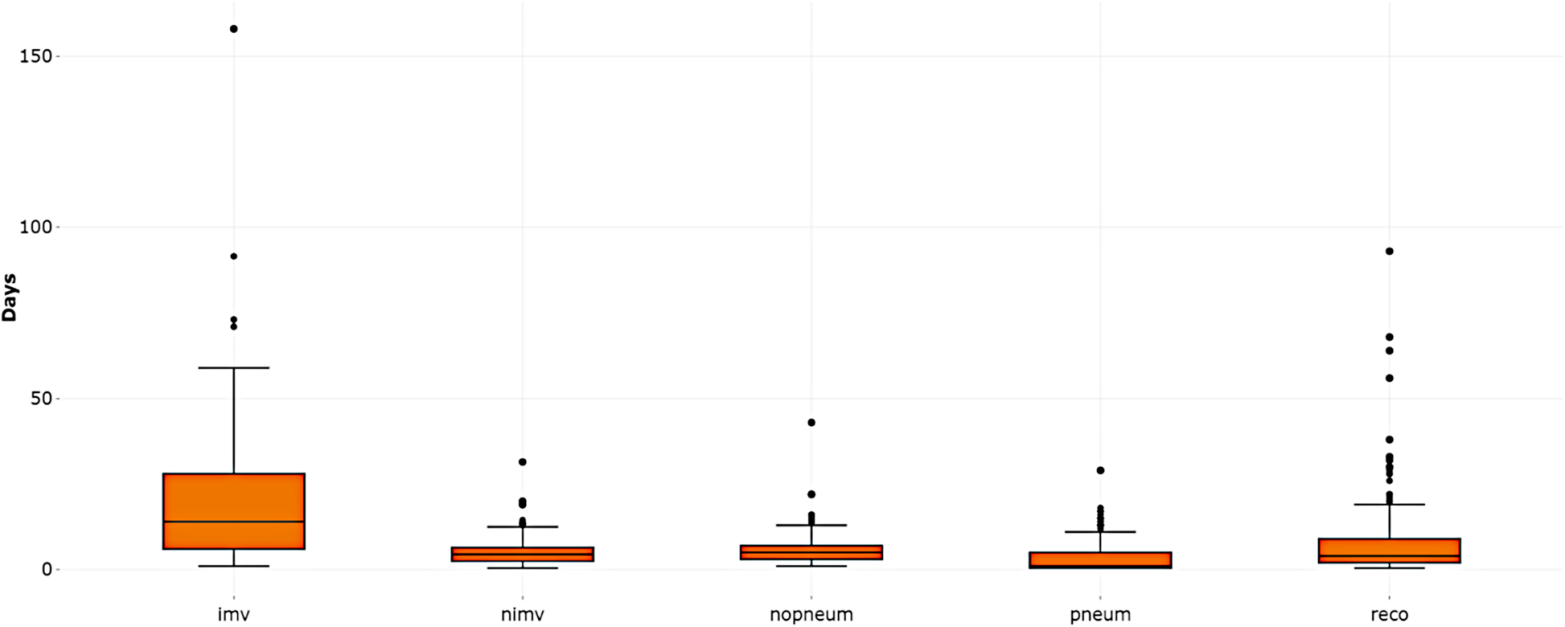
Length of stay in the initial/transient states

Box plots representing the length of stay in the initial or transient states of the model are a convenient tool, for uncensored data, to describe the distribution of these stays and to identify possible outliers before fitting the model. In Fig. 3, we can observe that the state with the highest median length of stay ($med=14 \; days$) is *imv*, while the other four initial/transient states (*nimv, nopneum, pneum, reco*) have lower and similar medians between them, all of them below 5. In our setting, it seems reasonable since *imv* is applied to patients in a critical situation that need longer times in that state to recover. Regarding to the outlier analysis, there are some patients that deserve more attention (e.g., a patient that has been in *reco* almost 100 days without being discharged).

In reference to the absorbing states the cumulative incidence plot is provided. It is useful to visualize the rate at which patients enter those states and the chances of reaching them in a given time. We observe that in the fifth wave approximately $5\%$ of the hospitalized COVID-19 patients died.

**Fig. 4:**
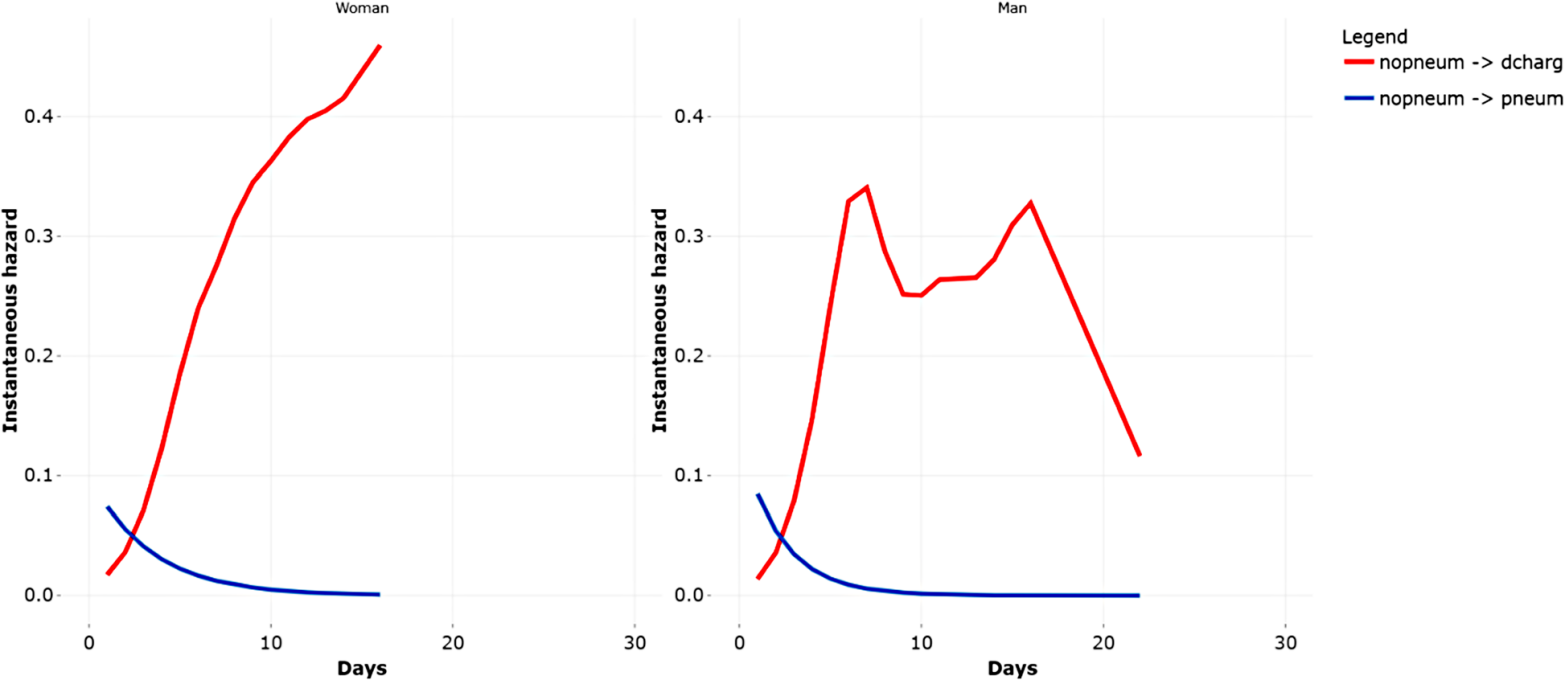
Exploring the data: instantaneous hazards stratified by sex

Finally, a plot with a non-parametric estimation of the instantaneous hazards of the transitions is presented, where data can be stratified by a single covariate (e.g.,*sex*, Fig. 4). For the transitions starting from the state no severe pneumonia, we can observe that women have an increasing risk of going from *nopneum* to *dcharg*, while they have a decreasing risk for transition *nopneum* $\rightarrow$ *pneum*. In the case of men, they have a decreasing risk for transition *nopneum* $\rightarrow$ *pneum*, but the risk for transition *nopneum* $\rightarrow$ *dcharg* changes over time. So, we conclude that there are not relevant differences according to the sex, and the more marked one is associated to the transition *nopneum* $\rightarrow$ *dcharg*, particularly after the seventh day.

### Model output

**Fig. 5:**
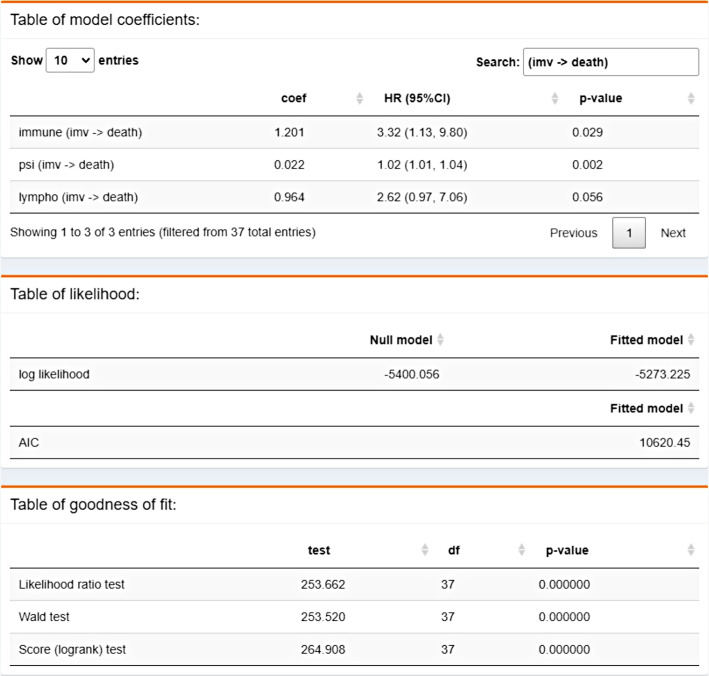
Tables of the fitted model

Markovian Cox models [1] can be selected from the drop-down menu within the **fitted model** tab. Other models are postponed for future releases. The model is fitted including all the previously selected transition specific covariates. The transition specific covariate selection was done based on background knowledge. After fitting the model three tables are returned giving different information of the model (Fig. 5).

**1) Table of model coefficients**. Each row represents a transition for a given covariate named in the first column of the table as *covar (*$k \rightarrow l$*)* where *covar* indicates the name of the covariate and $k \rightarrow l$ the transition of interest. For each covariate and transition the estimated coefficient (*coef*), the estimated hazard ratio, its confidence interval (*HR (95%CI)*), and the *p*-value (*p-value*) to test $\beta _{kl}=0$ for the corresponding covariate are provided. Those values indicate which is the association level of the covariate *covar* on the risk of having a specific transition.

**2) Table of likelihood**. It contains the values of the log-likelihood of the fitted and the null model. If a null model is fitted, the values of both log-likelihoods match. This table also provides the Akaike Information Criteria (AIC) [15] that allows to compare non-nested models.

**3) Table of goodness of fit**. The likelihood ratio, Wald, and score tests (*test*) are implemented to assess the goodness of fit of the proposed model versus the null model. For each test, the value of the statistic, the degrees of freedom (*df*) and the *p*-value are reported. These tests indicate whether or not the proposed model is more likely than the null model.

The estimated hazard ratios and their 95% confidence intervals are represented by forest plots to facilitate the interpretation of the risks associated with the different covariates in each transition. As the MSMs usually have too many coefficients, only the hazard ratios of the covariates related with a specific selected transition are shown. To accommodate covariates measured in different units, the user can scale the *effect* of any numerical covariate, for instance, by representing the risk related to 10-units change instead of 1 unit.

**Fig. 6:**
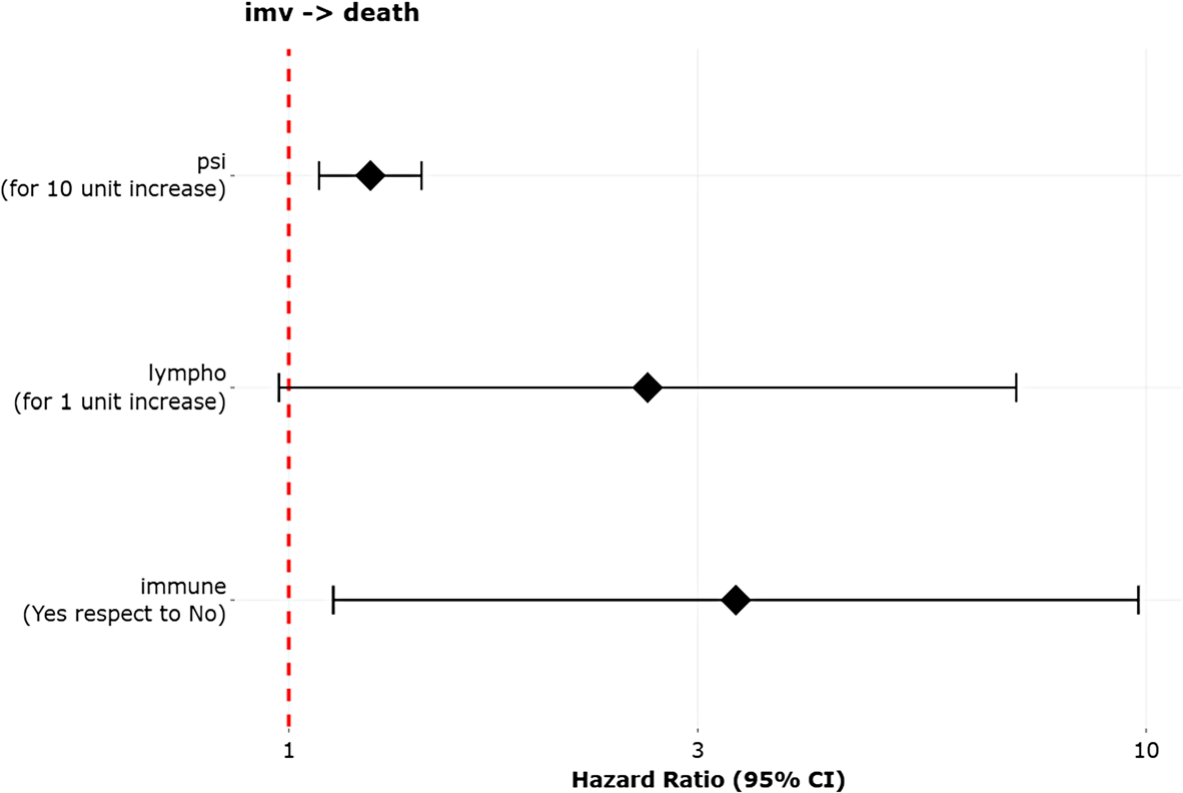
Forest plot of the covariates related with the transition imv $\rightarrow$ death

Figure 6 reveals that *psi* and *immune* are the covariates that have an *effect* on transition $\textit{imv} \rightarrow \textit{death}$: the risk of transitioning from invasive mechanical ventilation to dying increases 1.25 times when the pneumonia severity index of the patient increases 10 units, and it increases 3.32 times for immunosuppressed patients compared to non-immunosuppressed patients. There is no evidence to claim that the other covariates have an influence among those patients that transition from invasive mechanical ventilation to dying.

Some assumptions are made when fitting the Cox model and they should be evaluated for each transition after every preliminary fitting.

**Fig. 7:**
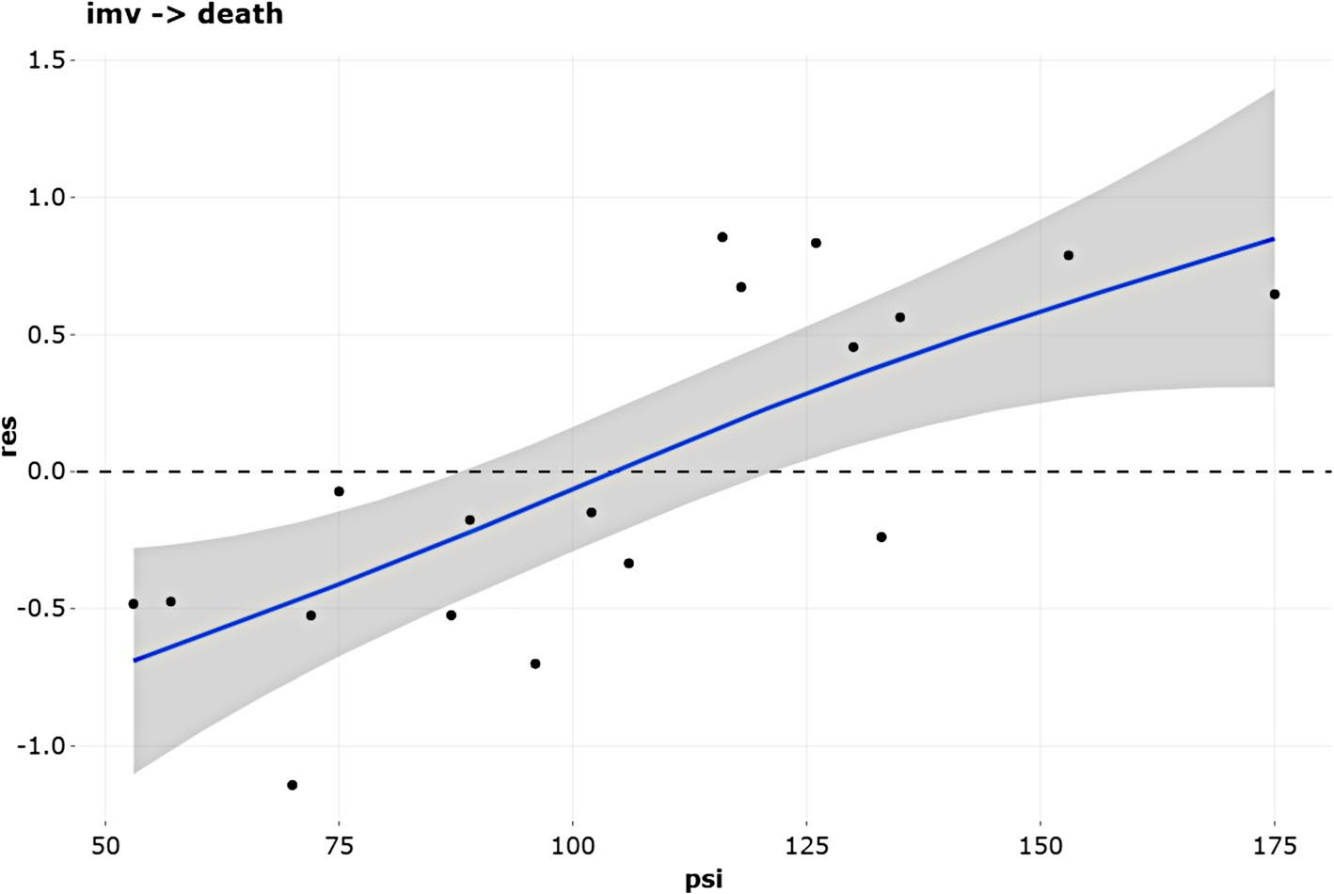
Validation of the assumption of linearity of the psi continuous covariate in the transition imv $\rightarrow$ death

Linearity of the numerical covariates is the first assumption to be assessed using martingale-based residuals. The blue smoothed curve represents a non-parametric estimate of the trajectory of the points along the covariate values indicating whether it is reasonably linear or not. In the case of the covariate *psi* and transition $\textit{imv} \rightarrow \textit{death}$ (Fig. 7), this premise can be assumed.

**Fig. 8:**
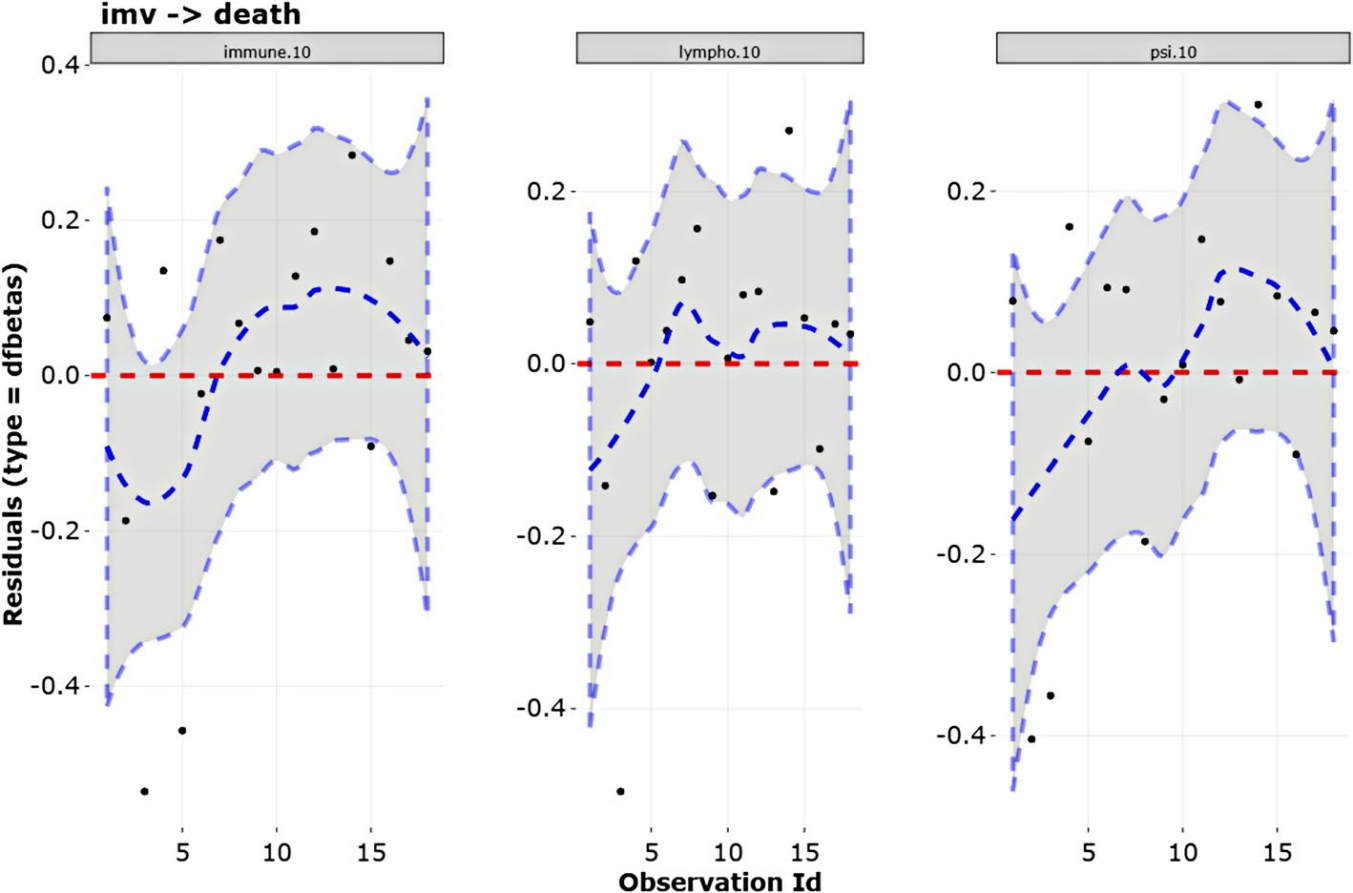
Validation of the assumption of absence of influential observations of covariates related with the transition imv $\rightarrow$ death

The second assumption, absence of influential observations, is analysed by the dfbetas residuals. When there are no points quite far from the others, it suggest the lack of influential observations(see transition $\textit{imv} \rightarrow \textit{death}$ in Fig. 8).

**Fig. 9:**
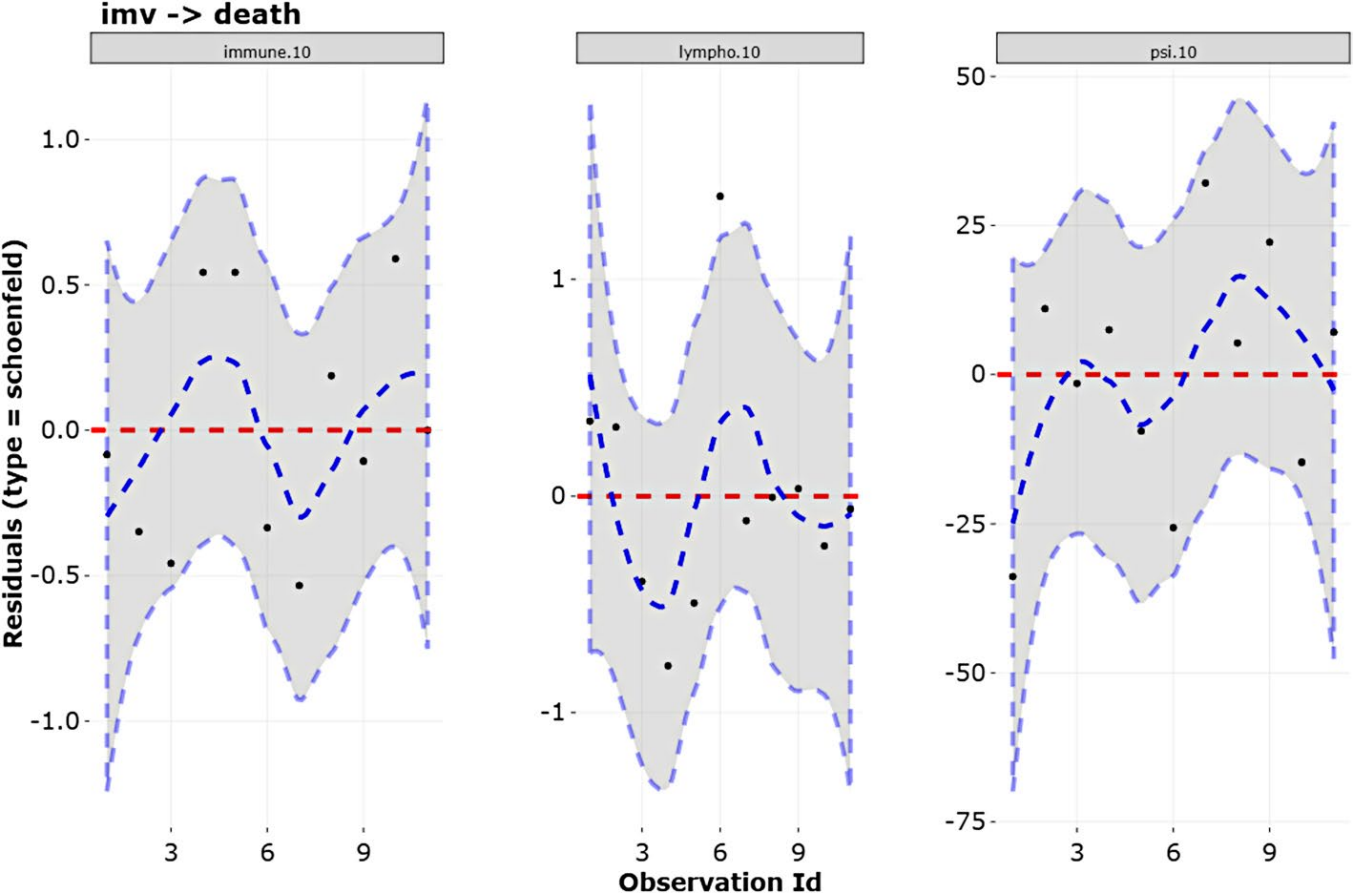
Validation of the assumption of proportionality of the hazards of covariates related with the transition imv $\rightarrow$ death

The last assumption, proportionality of the hazards, is assessed through the Schoenfeld residuals: the confidence bands of the smoothed curves should completely cover the line at 0. In Fig. 9, we see that this premise holds for transition $\textit{imv} \rightarrow \textit{death}$.

As all the assumptions hold for transition $\textit{imv} \rightarrow \textit{death}$, it is not necessary to go back and redefine the model fitting for this transition. But, before drawing conclusions or making predictions based on that model it is necessary to analyse every transition of the model.

As the fitted model will be used to predict, it is important to evaluate its global predictive performance. As the involved calculation has a high computational cost, this is only done on request by clicking the *predictive performance* button. Then, two tables will be showed: the first one returns the logarithmic score of the fitted model, while the second one represents the confusion matrix, containing the absolute frequencies according to the real (columns) and predicted (rows) states for a given follow-up time. Furthermore, a barplot representing the proportion of correct guesses for each state is provided.

In the **model comparison** tab, the information of each fitted model is stored in order to make possible their comparison. In addition, this information can be saved to be subsequently loaded in other session.

### Predictions

Predictions on new subjects as well as comparison of two subjects with different profiles are implemented in **MSMpred**. For instance, we want to contrast the evolution at 30 days of two hospitalized patients with non-invasive mechanical ventilation. They are both females, 80 years old, with a Charlson index and CURB-65 of 2, with a pneumonia severity index of 100, a C-reactive protein of 90 ng/ml and a safi and lymphocytes of 428.571 mmHg and $0.93 \times 10^3$ cells/$\text {mm}^3$, respectively. The first woman has cardiovascular as well as autoimmune diseases and she is not vaccinated against COVID-19. The second woman is vaccinated and does not have neither cardiovascular nor autoimmune diseases.

**Fig. 10:**
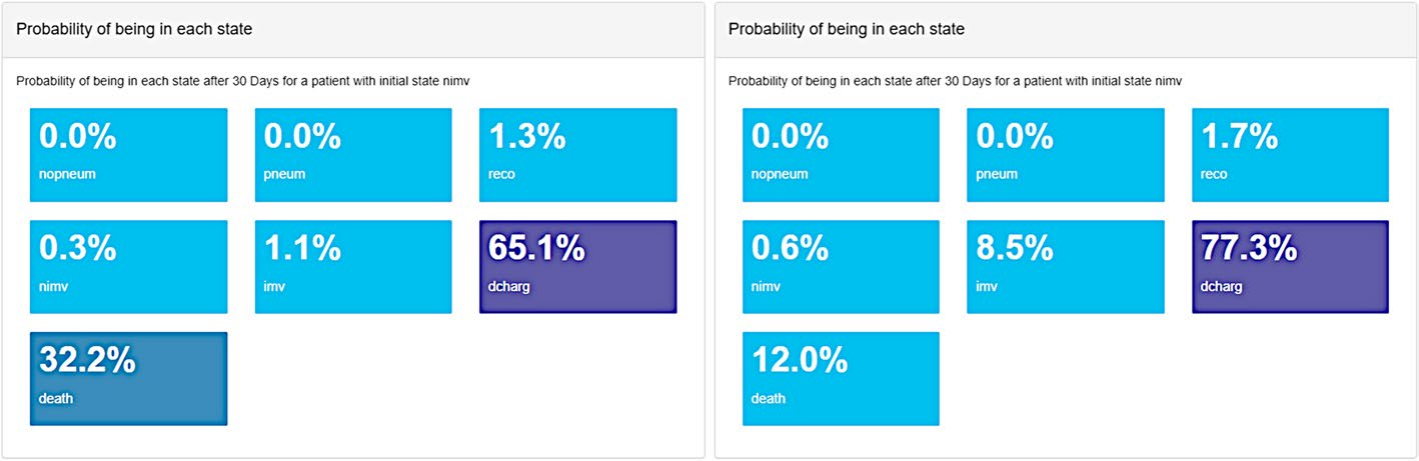
Predictions of the probability of being in each state after 30 days for the two new patients

Once the profiles have been defined, we obtain the probability of being in each state after 30 days for each patient (Fig. 10), regardless of previously visited states. We can observe that the evolution of first patient is less optimistic with 65.1% probability of having left the hospital before day 30 and 32.2% probability of dying, while the second one has a better prognosis with a probability of discharge before day 30 of 77.3%.

**Fig. 11:**
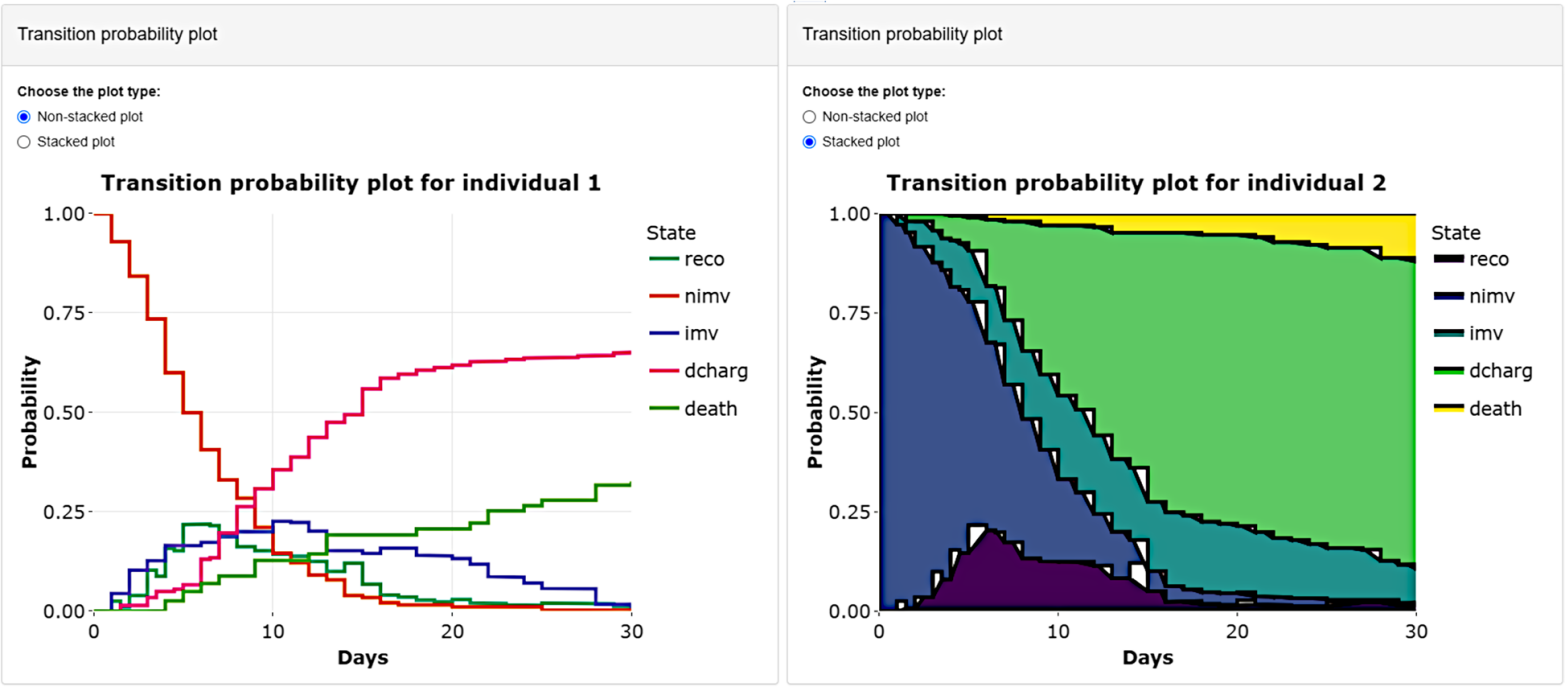
Transition probability plots for the two new patients, the first in a non-stacked way and the second in a stacked way

Graphically, Fig. 11 shows those percentages via transition probability plots, in a non-stacked or stacked way. Both versions give the probability of being in each state in a specific time point. In the case of the stacked plot, that probability is the height of the coloured section corresponding to the state of interest, while in the case of the non-stacked plot, it is directly the value of the curve of the state of interest.

## Discussion

**MSMpred** is a powerful tool to address a wide spectrum of problems. **MSMpred** can be used for modelling the course of any disease by means of MSM models (e.g., cancer, Alzheimer, fertility, chronic diabetic complications) with different degrees of severity. **MSMpred** is also appropriate in other fields outside the biomedical setting, for instance in multistate reliability assessment.

**MSMpred** allows to fit an MSM and to make predictions for new individuals in a friendly way providing several graphics that help interpreting those results.

Although other apps and packages to work with MSM exist, there are some unique characteristics of our app that make the difference. Regarding to the model definition, **MSMpred** does not limit the number of states or transitions: Furthermore, a diagram of the model is depicted while it is being specified. In order to build this diagram the number of states and transitions need to be taken into account. In addition, the user can delete transitions if by error a transition has been included or a transition is not needed. As seen, the covariates can be specified for each transition allowing a maximum level of detail in the model specification. Regarding visualization, several points are noteworthy. The instantaneous hazard plot, albeit common in this type of models, can be instantly obtained by specifying the ending/starting state of interest and also stratifying by some covariate. Furthermore, **MSMpred** shows the forest plot of the covariates related to the transition of interest. In relation to predictive performance, **MSMpred** provides outputs of the overall performance both for comparing models to each other (e.g., the logarithmic score) and more interpretable measures of accuracy for a single model (e.g., the confusion matrix). Finally, obtaining the validation graphics automatically allows the user to speed up model fitting with the corresponding saving of time in research work.

**MSMpred** is being constantly updated. In particular we are working on a new dataset including censored observations. We would also like to implement the option of including time-dependent covariates into the model, as well as allowing to fit other type of MSMs (e.g., non-Markov Cox models, semi-Markov models, parametric models). Finally, we are working in the development of a formal methodology to compare the goodness of fit of different MSMs.

## Conclusions

**MSMpred** is an R shiny app presenting useful tools for description, analysis, prediction and visualization of the different stages of a disease. **MSMpred** facilitates, in an interactive way, a better understanding of the potential paths along the states, of the risks associated to each transition and on the prognosis for a new subject. Non-experienced users might use **MSMpred** because is an intuitive and visual app and does not require neither programming skills nor advanced statistical knowledge. If needed, basic statistical knowledge on survival analysis can be obtained in classic references such as Klein [16]. For a thorough monograph on Cox Model we suggest [8].

## Data Availability

The example dataset of the DIVINE project supporting the conclusions of this article is not available due to confidentiality reasons, but a modified example dataset is available in the Github repository: https://github.com/LeireGarmendia/MSMpred. The detailed code of the app can be found in the same Github repository.

## Abbreviations

MSM: multistate model
DIVINE: Dynamic evaluation of COVID-19 clinical states and their prognostic factors to improve the intra-hospital patient management
ICU: Intensive Care Unit
NIMV: Non-Invasive Mechanical Ventilation
IMV: Invasive Mechanical Ventilation
$k \rightarrow l$: Direct transition from state *k* to state *l*
HR: Hazard ratio
LS: Logarithmic score

## Availability and requirements

Project name: MSMpred.

Project home page: https://www.grbio.eu/pubs/MSMpred/

Operating system(s): Platform independent.

Programming language: R.

Other requirements: Any web browser.

License: published under the GNU General Public License Version 2.

Any restrictions to use by non-academics: Commercial organizations are welcome to contact the author prior to use.
